# The diagnostic certainty levels of junior clinicians: A retrospective cohort study

**DOI:** 10.1177/18333583211019134

**Published:** 2021-06-11

**Authors:** Yang Chen, Myura Nagendran, Yakup Kilic, Dominic Cavlan, Adam Feather, Mark Westwood, Edward Rowland, Charles Gutteridge, Pier D Lambiase

**Affiliations:** 1 University College London, UK; 2 The London School of Economics and Political Science, UK; 3 St Bartholomew’s Hospital, 9744 Barts Health NHS Trust, UK; 4 4615 Imperial College London, UK.; 5 Royal London Hospital, 9744 Barts Health NHS Trust, UK

**Keywords:** decision certainty, decision confidence, clinical decision-making, electronic health records

## Abstract

**Background:**

Clinical decision-making is influenced by many factors, including clinicians’ perceptions of the certainty around what is the best course of action to pursue.

**Objective:**

To characterise the documentation of working diagnoses and the associated level of real-time certainty expressed by clinicians and to gauge patient opinion about the importance of research into clinician decision certainty.

**Method:**

This was a single-centre retrospective cohort study of non-consultant grade clinicians and their assessments of patients admitted from the emergency department between 01 March 2019 and 31 March 2019. De-identified electronic health record proformas were extracted that included the type of diagnosis documented and the certainty adjective used. Patient opinion was canvassed from a focus group.

**Results:**

During the study period, 850 clerking proformas were analysed; 420 presented a single diagnosis, while 430 presented multiple diagnoses. Of the 420 single diagnoses, 67 (16%) were documented as either a symptom or physical sign and 16 (4%) were laboratory-result-defined diagnoses. No uncertainty was expressed in 309 (74%) of the diagnoses. Of 430 multiple diagnoses, uncertainty was expressed in 346 (80%) compared to 84 (20%) in which no uncertainty was expressed. The patient focus group were unanimous in their support of this research.

**Conclusion:**

The documentation of working diagnoses is highly variable among non-consultant grade clinicians. In nearly three quarters of assessments with single diagnoses, no element of uncertainty was implied or quantified. More uncertainty was expressed in multiple diagnoses than single diagnoses.

**Implications:**

Increased standardisation of documentation will help future studies to better analyse and quantify diagnostic certainty in both single and multiple working diagnoses. This could lead to subsequent examination of their association with important process or clinical outcome measures.

## Introduction

Clinical medicine is characterised by uncertainty: patients present and manifest pathology in a myriad of ways. Most clinical research focuses on creating an evidence base to support the safe and effective use of new or existing diagnostics and treatments. However, the decision-making processes required to select the optimal clinical strategy have not been assessed as rigorously. Accurate decision-making represents a key step in providing high-quality healthcare to patients (Saposnik et al., 2016). One important factor that may influence the decision to select a particular investigation or treatment is the degree of certainty a clinician feels regarding his or her working diagnosis. Such certainty may be affected by a multitude of both internal and external factors as well as a lack of complete data when making decisions (Simpkin and Schwartzstein, 2016).

The act of diagnostic calibration is the process by which a clinicians’ confidence in the accuracy of their diagnosis aligns with their actual accuracy (Meyer et al., 2013, 2015; Meyer and Singh, 2019). This alignment of confidence and accuracy requires that both are precisely measured. For the former, the explicit measurement of confidence or certainty has been predominantly assessed in controlled environments, such as retrospective questionnaires after the clinical interaction, vignette studies or in simulations (Baldwin et al., 2005; Lawton et al., 2019; Logan, 2000; Yee et al., 2014). Better alignment of confidence and accuracy could mitigate against errors related to hubris or devaluing of underconfident opinions (Treadway, 2018), and in a 2015 Institute of Medicine report, it was noted that “nearly all patients will experience a diagnostic error in their lifetime, sometimes with devastating consequences” (McGlynn et al., 2015).

A recent systematic review focusing solely on the certainty of clinical decision-making in real time captured only nine studies – all of which used a measurement tool such as a Likert or visual analogue scale (Nagendran et al., 2019). Our study therefore had two primary research aims: to characterise the documentation of diagnoses by clinicians in a real-world setting of an acute medical admission; and to highlight the distribution of self-rated certainty among documented diagnoses and to suggest relevant follow-on research questions to test hypotheses generated as a result of this study. A secondary aim of our study was to obtain qualitative and quantitative feedback from a group of patients on the importance of research into decision certainty by clinicians.

## Method

The study protocol was registered with the Clinical Effectiveness Unit at Barts Health NHS Trust (ID no. 10201). Permission from the Chief Clinical Information Officer was granted to conduct a service evaluation project assessing the documentation of patients admitted via the acute medical team at the Royal London Hospital (RLH) by clinicians using an electronic health record (EHR), called Cerner Millennium. All data were stored on a secure server used by Barts Health NHS Trust for quality improvement and research studies. The article has been prepared according to The Strengthening the Reporting of Observational Studies in Epidemiology (STROBE) Statement (von Elm, 2007).

### Patient and public involvement

Prior to the main research study commencing, in March 2019, a patient focus group was convened to assess the views of patients about the importance of investigating clinicians’ diagnostic certainty levels. Advertisements were sent via a patient charity (Arrhythmia Alliance) and patients volunteered to be part of the event. Eleven (11) patients (7 female and 4 male) attended. Pre and post an information and discussion session lasting approximately one-and-a-quarter hours, patients were asked a single question: “How important to patients and the public do you think it is to conduct this research?” This session was not mandated as part of the service evaluation and therefore was designed as an informal assessment without the use of validated questionnaires, rather than a full-scale piece of qualitative research. The responses were completed anonymously on a simple five-point Likert-type scale that ranged from 1 (*not at all important*) to 5 (*very important*).

### Data sources

For the main study, a standardised proforma was used by all clinicians when assessing patients referred to the medical team (clerking proforma). These were patients who had been referred to the medical team by the emergency department (ED) after initial triage and assessment. Acute medicine clinicians had access to the initial assessment documentation of the ED team (which was not evaluated during this study).

During the data collection period, use of the clerking proforma was mandated as part of local departmental governance at the RLH. EHR data at the RLH can be accessed by Cerner Business Objects – a service evaluation tool used by Barts Health NHS Trust (Cerner, 2020) – which provides a graphical user interface to analyse data within the EHR and run SQL queries. We extracted all clerking proformas recorded in March 2019 and cross-checked the list against a manual database of admissions maintained by the acute medicine department. Extracted clerking proformas were anonymised and then exported to Microsoft Excel where a regular expression function was used to crop the working diagnosis from each clerking proforma. Granular data regarding the clinicians who were working at the hospital were not collected. However, an overview of the grades of clinicians during the study period was available, ranging between doctors with more than 1-year post-qualification experience (i.e. “core level doctors” and in UK system: Foundation Year Two, Core Medical Trainees/Internal Medicine Trainees, GP Specialty Trainee, Acute Care Common Stem trainees or other locally employed core level equivalent Doctors) to those with more than 5 years of post-qualification experience (Specialty Trainees or other locally employed specialty level equivalent).

### Hierarchy of certainty, definition of working diagnosis and diagnosis type

**Figure 1.**
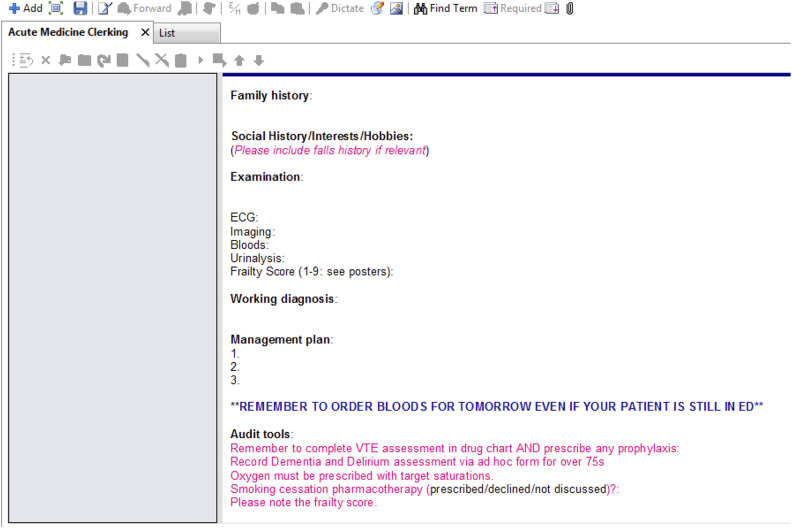
Appearance of clerking proforma to clinicians at study site.

**Figure 2.**
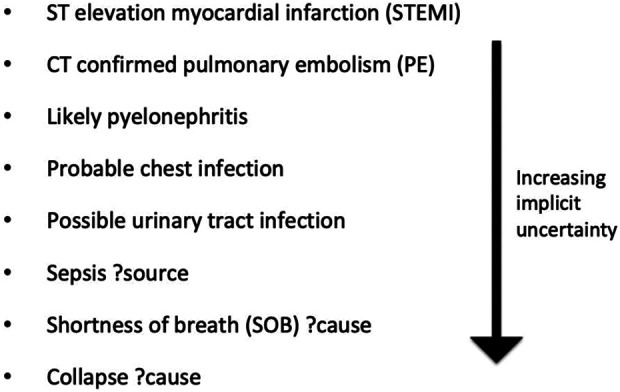
Examples of terms used in study sample and associated ranking of certainty.

Two clinicians, each with a 7-year postgraduate clinical experience (YC and MN), along with expert input from three clinicians with >50 years combined postgraduate clinical experience (DC, AF and PDL) agreed upon the classification of terms documented in the “working diagnosis” field of the clerking proforma, shown in Figure 1. Figure 2 shows examples of terms used in the study sample and their associated level of certainty. A diagnosis was defined as whatever the clinician had documented at the time of assessment in the “working diagnosis” section and was subject to patient factors (complexity of the case) as well as individual factors (documentation style). Many were, strictly speaking, not clinical diagnoses in the traditional medical sense; some were even repetitions of the presenting complaint (see results section for details).

All “working diagnoses” were split into two initial categories: single diagnoses and multiple diagnoses. For single diagnoses, categorisation of certainty was made on the basis of the specific adjectives or descriptors present. This categorisation was modelled on high stakes decision-making in the face of uncertainty in other settings – based on the National Intelligence Community in the United States (Office of Director of National Intelligence, 2015) (see Box 1). Single diagnoses were also subdivided based on the hierarchy of diagnosis level. For example, the lowest level was a symptom or sign-based diagnosis (e.g. chest pain). In cases where the documented diagnosis was repeating a patients’ reported chest pain, the expectation was that this would carry no certainty descriptor or for certainty to be definite. The level above this was a diagnosis based on a laboratory result (e.g. hyperkalaemia). In such cases, the expectation was once again that this would carry no certainty descriptor or for certainty to be definite. These two levels were contrasted against clinical diagnoses of conditions (e.g. unstable angina, where the expectation was that a certainty qualifier would be attached). For multiple diagnoses, a more basic analysis was conducted, after discussion between the authorship group revealed differences in the interpretation of the written record and difficulties in allocating such records to certainty descriptors for each diagnosis. It was recognised that this group of results was at particular high risk of bias. Box 2 outlines examples of these complex records:

Box 1. Categorisation of certainty based on National Intelligence Community in the United States.

**Table:** 

	No qualifier	Definitely not	Very unlikely	Less likely	Likely	Very likely	Definite
Synonyms	n/a	Excluded	Doesn’t, rule out, need to exclude	?, Query, Possible, Consider	Probable	More likely	Confirmed
% likelihood of stated condition	n/a	0	0–20	20–55	55–80	80–99	100
Examples	Septic shock secondary to CAP NSTEMI	HIV excluded by negative serology Mumps excluded as has been vaccinated	Inflammatory disease, aetiology unclear. Rule out TB Need to exclude VP shunt infection	? viral illness Consider paracetamol overdose Possible subarachnoid haemorrhage	Likely gastritis causing vomiting Probable sepsis	Acute confusion, very likely to be either overdose of prescription medication or recreational drug use	Confirmed PE on CTPA Definite STEMI

CAP: community acquired pneumonia; TB: tuberculosis; VP: ventriculo-peritoneal; CTPA: computed tomography pulmonary angiogram; PE: pulmonary embolus; HIV: human immunodeficiency virus; NSTEMI: non-ST segment elevation myocardial infarction.

Box 2. Examples of complexity of documentation of multiple diagnoses on the clerking proforma.

**Table:** 

Sample working diagnosis from assessments	Are diagnoses separate or are some layered/differentials/contain triggers or sequelae?	Was uncertainty expressed?
Reduced GCS 9/15 – ? cause,? medication-related – coincided with clozapine Hypertension – BP ∼200 systolic Less likely neuroleptic malignant syndrome – rare on clozapine, high creatine kinases but no fever/rigidity/raised WCC	Some separate diagnoses, some are potential differentials	Yes
Fall – multifactorial poorly controlled Parkinsons deterioration in patient’s mobility catheter associated UTI	Some separate	No
LRTI on B/G of COPD Multifactorial fall due to postural hypotension (secondary to drugs and dehydration), frailty --> need to rule out silent cardiac event as patient has had a history of this. --> not in keeping with aortic stenosis being cause of syncope AKI on CKD likely due to poor oral intake	Some separate	Yes
HAP Frailty Poor oral intake – dry	Separate, but some descriptors may relate to co-morbidities and background	No
Difficult diagnosis Most likely sepsis? source with high lactate Chest, abdominal, CNS Acute behavioural disturbance, on antipsychotics, no obvious history of substance abuse Neuroleptic malignant syndrome – essentially normal peripheral neurological examination – unlikely	Some separate, some are potential differentials	Yes

GCS: Glasgow coma score; BP: blood pressure; WCC: white cell count; UTI: urinary tract infection; COPD: chronic obstructive pulmonary disease; AKI: acute kidney injury; CKD: chronic kidney disease; HAP: hospital acquired pneumonia; CNS: central nervous system.

Thus, multiple diagnoses either represented more than one condition being manifest in that particular clinical case or represented the explicit recognition of diagnostic uncertainty through differential diagnoses. Additionally, some records pertained to layering of diagnoses – for instance secondary diagnoses that were sequelae or triggers of the main diagnosis. These also proved difficult to analyse in a systematic manner. Therefore, we categorised multiple diagnoses as either containing any expression of uncertainty within them or not.

## Results

From the patient and public involvement (PPI) work, a mean score of 4.5/5 and 4.9/5 were recorded at the start and end of the focus group session, in response to the question “how important to patients and the public do you think it is to conduct this research?” There was unanimous verbal and written support for more research to be conducted on the topic of uncertainty in working diagnoses for medical patients, with agreement that low certainty ratings could help identify difficult cases and promote greater collaboration.

**Figure 3.**
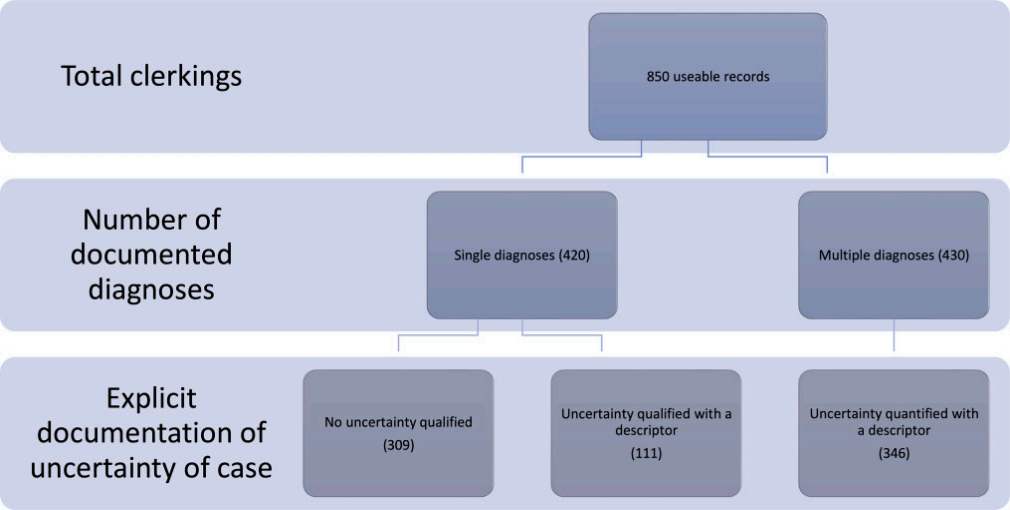
Summary of documentation of assessment, categorised by number of diagnoses.

In all, 865 clerking proformas were analysed. Automated data extraction was 100% complete when cross-referencing against the manual database. Fifteen (15) records were unsuitable for further analysis (9 had no diagnosis recorded, 3 could not be extracted from the EHR due to technical issues and 3 were elective as opposed to acute admissions). This left 850 available for further analysis of which 420 presented a single diagnosis while 430 presented multiple diagnoses (see Figure 3).

Of the 420 single diagnoses, 67 (16%) were symptom or sign-defined while 16 (4%) were laboratory-result-defined diagnoses. The remaining 337 (80%) were diagnoses of a specific condition. There was no adjective or quantification of uncertainty in 74% of the single diagnoses (309 of 420) and no diagnosis was described as definite, confirmed or excluded. The description of certainty in the remaining 111 cases was categorised according to the classification system in Box 1 as follows: *very unlikely* (16), *less likely* (58), *likely* (31), *very likely* (6).

Of the 430 multiple diagnoses, uncertainty was expressed in 346 (80%) compared to 84 (20%) in which no uncertainty in the diagnoses was expressed. In total, 71 different clinicians contributed to the assessment of medical patients during the study period. Their names and grades were not available for analysis as part of this service evaluation; however, the clinical staffing model at RLH would allow for an approximation of a 4:1 ratio of clinicians with between 1 year and 4 years of postgraduate experience to clinicians with 5+ years of postgraduate experience.

## Discussion

To the best of our knowledge, this study is the first real-world analysis of how uncertainty in the working diagnosis is documented at an acute medical hospital in the United Kingdom. There are four key findings:

1. Approximately half of working diagnoses contained only a single diagnosis as opposed to a differential.
2. In nearly three quarters of single diagnoses, no element of uncertainty was documented – this may relate to the way UK clinicians are trained or may reflect cases that are straightforward.
3. A fifth of single diagnoses were defined entirely by a symptom/physical sign or laboratory result rather than generation of an actual clinical diagnosis.
4. Characterising multiple diagnoses into more granular categories was predictably difficult, owing to the inherently varied ways in which they were recorded. The recording of uncertainty among non-consultant grade clinicians appears ad hoc, implicit and discretionary, making quantitative analysis and designing future quality improvement efforts difficult. This does not, however, mean that such work is not important. On the contrary, quantification may speak to the good handover of information to colleagues which maintains patient safety and mitigates against the degradation of information over time during a patient’s admission.

### Comparison with the literature

Decision-making research has focused on surveys undertaken away from the frontline or simulated scenarios using case vignettes (Blumenthal-Barby and Krieger, 2015) – this allows for careful control of multiple variables that may influence the results. The paucity of research examining decision-making in real-world settings may be due to a perceived lack of ability to control for obvious variables and to be able to meaningfully link a snapshot decision to clinical outcomes such as length of stay or inpatient mortality. However, our contention is that there is value in pursuing this line of research even if it is difficult. There is a number of beneficial second-order effects that could arise through more robust and meaningful pursuit of analysing real-world decision-making. For example, there may be educational value, both for trainees and senior doctors – and the wider workforce – in seeing how their self-rated certainty relates to longer-term outcomes, along with how they compare to their peers. There is evidence that already exists for the benefits of an embedded audit and feedback system that facilitates reflective practice for staff (Patel et al., 2018).

In a detailed mixed methods study by O’Hara et al (2014), staff raised concerns regarding the impact of external pressures on decision-making such as admission avoidance guidance: “You are almost going into a ridiculous level of risk management, which actually isn’t to do with patient care.” The study of decision-making and how it relates to internal and external pressures will rely upon more systematic measurement of decision-making certainty, which is currently open to interpretation – the meaning of *likely* or *probable* will be different for different people and has been the subject of decades of behavioural science, sociology and psychology research (Tetlock and Gardner, 2015).

### Follow-up

There are three parallel questions that emerge from this study:

1. Can we prospectively measure certainty levels in a more robust manner and are they associated with process and outcome measures?
2. To what extent is certainty attributable to the patient versus hospital-specific or clinician factors (e.g. simple vs complex patient, a night vs day shift, a clinicians’ seniority or even their personality as it relates to thought processes?)
3. Can we design cognitive interventions to mitigate against miscalibration between diagnostic accuracy and diagnostic confidence?

One potential next step would be to introduce a Likert scale within the EHR asking the clinician to rate their certainty level for the working diagnosis. Such quantification would allow easier testing of pragmatic research questions, though care will be needed to interpret situations when diagnoses overlap or outcomes and management strategies relate to one another (Assel et al., 2017).

We recognise that even quantification would retain a subjective element. One doctors’ perception of 90% certainty will be different to that of another. This is unavoidable and quantitative ratings would nonetheless contain valuable insights. Adding an explicit “self-rated certainty field” to be completed by clinicians at the time of documentation could introduce a Hawthorne effect, particularly if clinicians were aware that their documentation was being analysed as part of a research study. On balance, embedding a self-rating field should at least be considered in spite of such biases, given the rich data that could be generated and the potential for safety gains by improving the quality of information handed over. For example, inserting the following label “Certainty in working diagnosis from *low* [1] to *high* [10]” beneath the “Working diagnosis” heading on future clerking proformas.

In any future work, care must be taken not to add any further burden onto the already stretched clinical workflow. Bassford et al. (2019) demonstrated that adding a standardised referral form when deciding to admit a patient to intensive care had barriers to uptake. However, of the one-third of clinicians who made use of the form, there was evidence supporting an impact on decision-making: clinicians noted that the forms had prompted them to consider blind spots in their thinking including a greater focus on the views of the patient.

### Implications

Explicit self-rating of certainty could directly contribute to improving patient safety and advocacy through the Hawthorne effect. The mere act of quantifying a certainty rating might form a cognitive brake and thus an important debiasing strategy that mitigates against avoidable medical error occurring from under- or overconfidence (Croskerry et al., 2013a, 2013b; Mamede et al., 2010a, 2010b; Topol, 2019). Patients in our PPI work agreed that low certainty ratings could help identify difficult cases and prompt greater teamwork. It is conceivable that improvements in clinical processes such as reduced waste from over-investigation or better patient flow to appropriate environments could arise as a result of highlighting patients where decisions are made with high or low certainty.

More broadly, in a systematic review of real-time decision-making (Nagendran et al., 2019), one study reported that a high uncertainty for the diagnosis of heart failure was associated with a longer length of stay, increased mortality and higher readmission rates at 1 year. Future research will likely examine prospective testing of artificial intelligence (AI) decision support tools in healthcare and a logical question to test is how clinicians will interact and engage with such tools (i.e. what factors (including certainty levels) influence whether clinicians agree or disagree with AI-recommended management decisions).

### Limitations

Our findings must also be considered in the light of several limitations. First, we were unable to link the working diagnosis and the final discharge diagnosis to assess how documented uncertainty relates to diagnostic accuracy. One aspect of changing working patterns in modern medicine has been the increase in handovers of care. Diagnostic labels made by one clinician may often be carried over by another, and one further area for future research is the degree of “copy and paste” entries that can increase the possibility of clinicians being susceptible to anchoring and other cognitive biases. Of note, while clinical coders are tasked with assigning codes based on documentation, discharge summaries would be written with the same risk of inherent variability in practice and documentation quality as observed with clerking proformas. There are many examples of variation in discharge summary documentation from the literature, including an analysis from the United States that examined the question in the specific setting of heart failure (Al-Damluji, 2015). Thus, the effect of unclear diagnoses and imprecise clinical documentation on coding is considerable – notwithstanding the impact on national data collection and reimbursement, there could be exacerbation of idiosyncracies in coding convention that could further bias the analyses. For example, clinical coding (at least in the United Kingdom) is constrained by “probable” being an acceptable term whereas terms such as “likely” and “query” are not (DGCS.2, 2017). Second, we were not able to analyse the multiple diagnoses with the same granularity as the single diagnoses. We therefore made the pragmatic decision to approximate uncertainty by tagging the record as uncertain if there were any of the descriptors from Box 1 present. This may explain why a larger proportion of multiple diagnoses contained some degree of uncertainty. Third, we did not explore other relevant external factors that may have influenced decision certainty, including the time of day and seniority of clinician – the latter being a factor identified in a vignette study (Lawton et al., 2019) as a surrogate for greater tolerance of uncertainty in an ED setting. In our study, the pool of doctors varied in experience from 1-year postgraduate to as many as 10 years of experience. Collecting more granular information, including the timestamp of when clerking proformas were submitted and the stage of training and specialties of clinicians involved, represents crucial follow-on work. This could lead to hypothesis testing studies in which sample size calculations could be used to inform study design. Larger sample sizes as part of a “Learning Health System” (Etheredge, 2007) geared for research could examine additional associations, such as how clinician personality and risk-tolerance traits relate to real-time documentation of uncertainty. Other testable factors could also include the complexity of the patient and their acuity of presentation, as well as the comprehensiveness of the initial ED referral and assessment. Finally, the sample arises from a single teaching hospital and may not be representative of other institutions, particularly in international settings where educational or cultural differences may have a significant impact on clinician self-rating of certainty.

## Conclusions

In nearly three quarters of single diagnoses, no element of uncertainty is portrayed or quantified. Greater uncertainty is expressed in multiple diagnoses than single diagnoses. These data have implications for the design of prospective studies looking to assess how uncertainty is recorded and whether there is an association between certainty of working diagnosis and process measures or outcomes. Our PPI work highlighted how important this topic was for patients and there are additional factors to consider that could appear as emergent phenomenon when conducting future studies. These include the educational value to clinicians of linking their initial and discharge diagnoses and receiving tailored feedback, as well as the system-wide effects of better clinical documentation, such as more accurate clinical coding and routine data being of research-grade quality.
